# Identification of Biomarkers Associated With CD8+ T Cells in Coronary Artery Disease and Their Pan-Cancer Analysis

**DOI:** 10.3389/fimmu.2022.876616

**Published:** 2022-06-21

**Authors:** Shijian Zhao, Yinteng Wu, Yantao Wei, Xiaoyu Xu, Jialin Zheng

**Affiliations:** ^1^ Department of Cardiology, the Affiliated Cardiovascular Hospital of Kunming Medical University (Fuwai Yunnan Cardiovascular Hospital), Kunming, China; ^2^ Department of Orthopedic and Trauma Surgery, the First Affiliated Hospital, Guangxi Medical University, Nanning, China

**Keywords:** coronary artery disease (CAD), CD8+ T cells, hub genes, weighted gene co-expression network analysis (WGCNA), CIBERSORT, cancer

## Abstract

**Purpose:**

To identify biomarkers associated with CD8+ T cells in coronary artery disease (CAD) and initially explore their potential role in the tumor immune microenvironment.

**Materials and Methods:**

CAD-related datasets GSE12288, GSE34198, and GSE66360, were downloaded from the GEO database. First, GSVA was performed based on the GSE12288 dataset. Then WGCNA analysis was performed to identify the most relevant module and candidate hub gene for CD8+ T cells, followed by GO and KEGG analysis of this module. Secondly, the relationship between candidate hub genes and CD8+ T cells was verified using GSE34198 and GSE66360, which led to the identification of hub genes. The relationship of hub genes with CD8+ T cells in cancer was analyzed using the TIMER database. Methylation analysis of hub genes was performed using the DiseaseMeth database. CAD, pan-cancer, pan-cell lines, and pan-normal tissues, correlations between hub genes. In addition, potential drugs and TFs associated with hub genes were predicted, and the ceRNA network was constructed. Finally, GSEA was performed separately for hub genes.

**Results:**

CAD was shown to be associated with immune response by GSVA analysis. WGCNA identified the blue module as most related to CD8+ T cells and identified nine candidate hub genes. The relevance of CAD to immunity was further confirmed by GO and KEGG analysis of the module. Two additional datasets validated and identified three hub genes (FBXO7, RAD23A, and MKRN1) that significantly correlated with CD8+ T cells. In addition, we found that hub genes were positively associated with CD8+ T cells in TGCT, THCA, and KICH cancers by our analysis. Moreover, the hub gene was differentially methylated. We also analyzed the correlation between hub genes in CAD, different cancers, different cell lines, and different normal tissues. The results of all the analyses showed a positive correlation between them. Finally, we successfully constructed hub gene-associated TF-gene and ceRNA networks and predicted 11 drugs associated with hub genes. GSEA suggests that hub genes are related to multiple immune response processes.

**Conclusion:**

FBXO7, RAD23A, and MKRN1 are significantly associated with CD8+ T cells in CAD and multiple cancers and may act through immune responses in CAD and cancer.

## Introduction

Coronary atherosclerotic heart disease, or coronary artery disease (CAD), is the most common form of cardiovascular disease. CAD is the most common cause of human death, both domestically and abroad (1). To date, the gold standard for the diagnosis of CAD is the performance of coronary angiography, which has the highest accuracy. Still, this means of confirming the diagnosis is invasive and can only be done by cardiologists who have specialized studies in the cardiac intervention (2). Moreover, pharmacological treatment is essential throughout, in addition to medical intervention and surgical bypass surgery (3). In addition, cancer has a very high morbidity and mortality rate, and recent studies have found that cancer patients often have a combination of heart disease, especially CAD (4). However, little has been reported about the relationship between CAD and cancer. Therefore, the exploration of potential biomarkers associated with CAD and the exploration of possible mechanisms and common therapeutic targets between CAD and cancer deserve further investigation.

Atherosclerosis is the most basic pathological mechanism of CAD (5, 6). The immune response plays an integral role in the formation as well as in the development of atherosclerosis (7, 8). Among them, T lymphocytes are the primary immune cell type in atherosclerotic plaques, with a predominance of cytotoxic CD8+ T cells (9, 10). Animal experiments have shown that cytotoxic CD8+ T cells promote the formation of necrotic cores and exacerbate the progression of unfavorable plaques (11). It has been found that antibody-mediated depletion of CD8+ T cells ameliorates atherosclerosis (11). Also, CD8+ T cells can induce apoptosis and inflammatory responses by secreting cytokines such as tumor necrosis factor-α and IFN-γ (12, 13). In addition, CD8+ T cell function is tightly controlled by immune checkpoint proteins. Regulators of immune checkpoint proteins, such as the E3-ligase Casitas B-cell lymphoma B (CBL-B), control the progression of human atheromatous plaques mainly by limiting the activation CD8+ T cells (14). In contrast, co-suppressors that inhibit immune checkpoints, such as cytotoxic T-lymphocyte-associated protein 4 (CTLA-4), programmed cell death protein 1 (PD-1), and programmed cell death one ligand 1 (PD-L1), can increase cytotoxic CD8+ T cells and thus aggravate atherosclerotic plaques (15). Tumor immunotherapy most notably enhances the activity of CD8+ T cells to obtain a sustained and effective anticancer response (16). In the tumor microenvironment (TME), CTLA-4 inhibits the initiation of initial T cells, while PD-1 and PD-L1 accelerate effector T cell depletion (17). Blocking CTLA-4 or PD-1 enhances CD8+ T cell-mediated immune responses (18). In addition, immunomodulatory antibodies or cancer vaccines can restart CD8+ T-cell activity (19). In conclusion, immunotherapy has been widely used in the clinical treatment of cancer but is limited to laboratory studies on CAD.

Bioinformatics analysis methods have been utilized to conduct relevant explorations to gain insight into the underlying pathophysiological mechanisms of diseases. Among them, weighted gene co-expression network analysis (WGCNA) is a widely used bioinformatics analysis tool that can mine the gene expression modules most relevant to the immune response to disease, from which hub genes can be further screened (20). On the other hand, CIBERSORT is another bioinformatics analysis method that can identify the type of immune cell composition (21). In recent years, the combined application of WGCNA and CIBERSORT to explore potential biomarkers has been commonly used in oncology. However, few studies have used it to investigate potential biomarkers associated with CAD immune responses, especially to explore biomarkers related to CD8+ T cells.

We used the Gene Expression Omnibus (GEO) database to download the gene expression dataset GSE12288 from patients with CAD and first analyzed the enrichment of all sample genes in this dataset using GSVA. This dataset was then analyzed using WGCNA to identify the modules most associated with CD8+ T cell infiltration levels and candidate hub genes. Then, GO, and KEGG analyses were performed on this module to confirm the association of CAD with CD8+ T cells. In addition, we further explored the relevance of these candidate hub genes to CD8+ T cells using other datasets, GSE34198 and GSE66360, thus identifying three hub genes in CAD with CD8+ T cells. Finally, we also explored their role in different cancer types. This is the first time we have used WGCNA combined with immune infiltration analysis to identify potential molecular markers associated with CD8+ T cells in CAD while further exploring their possible immune mechanisms in CAD and different cancers. This discovery may provide a valid theoretical basis for the future development of novel immunotherapies for CAD and cancer and provide new insights into the possible link between CAD and cancer.

## Materials and Methods

### Gene Set Variation Analysis

Gene Set Variation Analysis (GSVA) is a non-parametric, unsupervised analysis method (22). It evaluated the enrichment of different signaling pathways of genes in the GSE12288 dataset in different samples. Gene sets C2 (containing GO information) and C5 (containing KEGG information) were first downloaded using the msigdbr package. Finally, gene set variation analysis was performed using the limma and GSVA packages.

### Acquisition and Processing of Gene Expression Data

The GSE12288 dataset was retrieved and downloaded from Gene Expression Omnibus (GEO, http://www.ncbi.), containing 110 coronary heart disease samples and 112 standard human samples (23). The dataset was first normalized using the normalized between arrays function of the limma package. Then the conversion between probe IDs and gene symbols was performed according to the GPL96 platform information. Probes without gene symbols were removed, and for multiple probes corresponding to the same gene symbol, the average expression value of each search was taken. We use the coefficient of variation values to select the genes with the most significant variation, select genes with gene variation coefficients greater than 0.1, and then use these genes to construct the network.

### Evaluation of Infiltration of CAD Immune Cells

The CIBERSORT algorithm is often used to calculate the proportion of 22 immune cell types (21). To understand the infiltration of immune cells in the samples in the dataset GSE12288, we used the R package “CIBERSORT” to calculate the raw data of GSE12288.

### Construction of Co-Expression Network

Based on the expression of genes with coefficients of variation greater than 0.1 in the GSE12288 dataset, we used the R package “WGCNA” to construct a gene co-expression network (20). The steps are as follows: we first convert the expression levels of individual transcripts into a similarity matrix based on the Pearson correlation values after gene pairing. Then, the similarity matrix is transformed into an adjacency matrix according to the formula as amn=|cMn|β (cMn=Pearson correlation between paired genes; amn=adjacency relationship between paired genes). When the power of β = 3, the adjacency matrix can be transformed into a topological overlap matrix. Finally, we choose the module minimum cutoff value of 30 and use the bottom-up algorithm to divide the genes with similar expression patterns into different modules.

### Building Module Feature Relationships

Component analysis was performed for each module according to its characteristics. We used Pearson’s test to analyze the correlation between module features and T-cell subtypes. When p<0.05, we considered the module to have a significant correlation with T-cell subtypes. We selected the module with the highest correlation coefficient with CD8+ T cells as the hub module.

### Functional and Pathway Enrichment Analysis

To further elucidate the biological functions and signaling pathways of the identified genes in the hub module, we performed gene ontology (GO) analysis (24) and Kyoto Encyclopedia of Genes and Genomes (KEGG) pathway analysis (25) using the “cluster profile” R package (26). Among them, GO analysis includes molecular functions (MF), cellular components (CC), and biological processes (BP).

### Identification and Validation of Hub Genes

We selected candidate hub genes based on module connectivity and clinical trait relationships for each gene in the hub module. Module connectivity was defined as the absolute value of Pearson correlation between genes (module membership). Clinical trait relationship is defined as the total value of Pearson correlation between each gene and trait (gene significance). For candidate hub genes, we set Module Membership>0.8 and gene significance>0.5. To verify whether there exists a significant positive correlation between candidate hub genes and CD8+ T, and thus identify reliable hub genes. We downloaded the GSE34198 (27) and GSE66360 (28) datasets and calculated the CD8+ T cells in each specimen using the cybersport algorithm. Spearman correlation between CD8+ T cell infiltration level and hub gene expression was also calculated. We defined hub genes as candidate hub genes significantly correlated with CD8+ T cells in the GSE12288, GSE34198, and GSE66360 datasets. In addition, we further validated the Spearman correlation between hub gene expression levels and CD8+ T cell infiltration levels using the Tumor Immune Evaluation Resource (TIMER) database. We visualized the results with “ggplots2”.

### Hub Gene Methylation Levels

Methylation gives each CD8+ T cell subpopulation its unique epigenetic fingerprint. Thus these cells can rapidly produce corresponding effects when they reencounter cognate antigens. DiseaseMeth 2.0 database stores abnormal methylation data for various human diseases (29). Therefore, we used it to explore the relationship between FBXO7, RAD23A, and MKRN1, three hub genes expression, and methylation levels in CAD.

### Hub Gene Correlation

In addition to the GSE12288 dataset, we also used two other datasets, GSE34198 and GSE66360, to synthesize the correlation between hub genes and then used “ggplots2” to visualize the analysis results. To further demonstrate the correlation between hub genes, we also downloaded high-throughput expression data of cancer, cell lines, and normal tissues from three databases, including TCGA, CCLE, and GETx, respectively. By calculating the correlation between hub genes in different cancers, different cell lines, and different normal tissues. Finally, “ggplots2” was used for visualization.

### The Linkage Between Hub Genes

mRNA controls many critical biological processes, and the functions it performs are closely related to its localization in the cell. mRNA located database is a comprehensive and advanced model for accurate eukaryotic mRNA subcellular localization expression. We used it to analyze the location of these three hub genes in the cell. Transcription factors regulate transcriptional processes by recognizing specific DNA sequences, thus participating in various complex biological processes. The ChEA3 database integrates RNA-seq data from ENCODE, ReMap, GTEx, TCGA, and ARCHS4, using which common transcription factors for multiple genes can be predicted. Since the mechanism of the role of ceRNA in CAD is still unclear, we used the ENCORE database to predict the microRNA of HUB genes in reverse and the LncRNA of the common microRNA of HUB genes, and finally constructed the ceRNA network. In addition, we completed the GO annotation data required for three different aspects of GOSemSim from BP, CC, and MF levels of the corresponding species. The functional similarity among hub genes was searched for by comprehensively assessing the gene’s function in the three groups.

### Small Molecule Drug

The CMAP (Connectivity Map) database has the most comprehensive data on the transcriptome of drug interference therapies (30). We placed FBXO7, RAD23A, and MKRN1 into CMAP, defining a negative connectivity score as a potential therapeutic agent. Therefore, we used enrichment <-0.7 and P<0.0 1 for screening.

### Gene Set Enrichment Analysis

Gene set enrichment analysis (GSEA) can be used to clarify whether a set of gene sets is significantly different between two biological states (31). To further explore the potential mechanisms by which the three identified hub genes affect CAD, we performed a single-gene GSEA analysis for this purpose. In the GSE12288 dataset, according to the expression levels of hub genes, their correlation coefficients with other genes were calculated separately using spearman analysis, and the correlation coefficients were sorted as the input gene list of the clusterProfilerR package. The h.all.v7.4.sytmbols.gmt from the Molecular Signature Database (MSigDB) was selected as the reference gene set, and a P-regulation value <0.05 was used as the screening criterion.

## Results

### Gene Set Variation Analysis of Gene Expression Data

**Figure 1:**
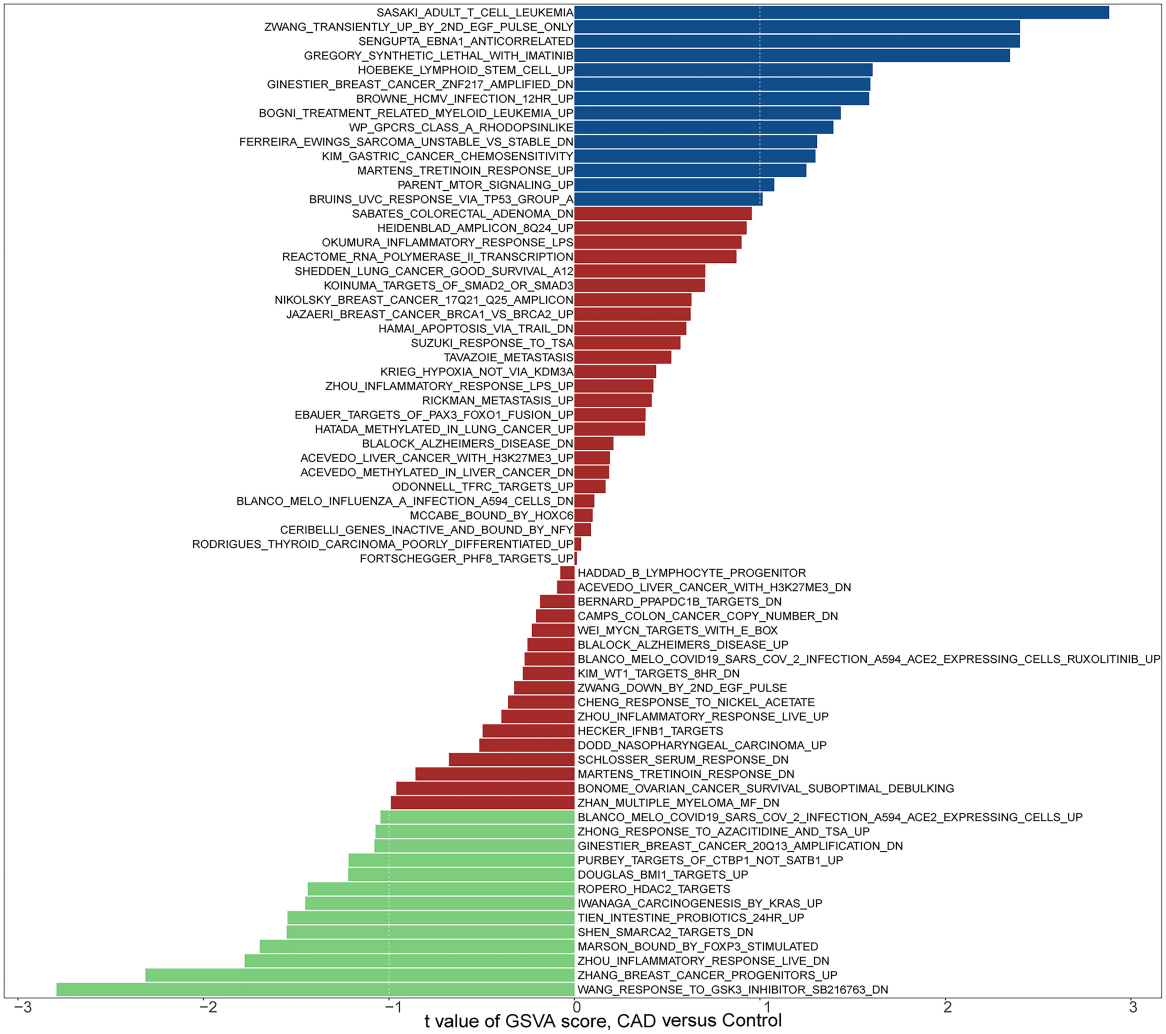
Biological processes and pathways associated with all sample genes in the GSE12288 dataset related to CAD. Hallmark section of GSVA.

**Figure 2:**
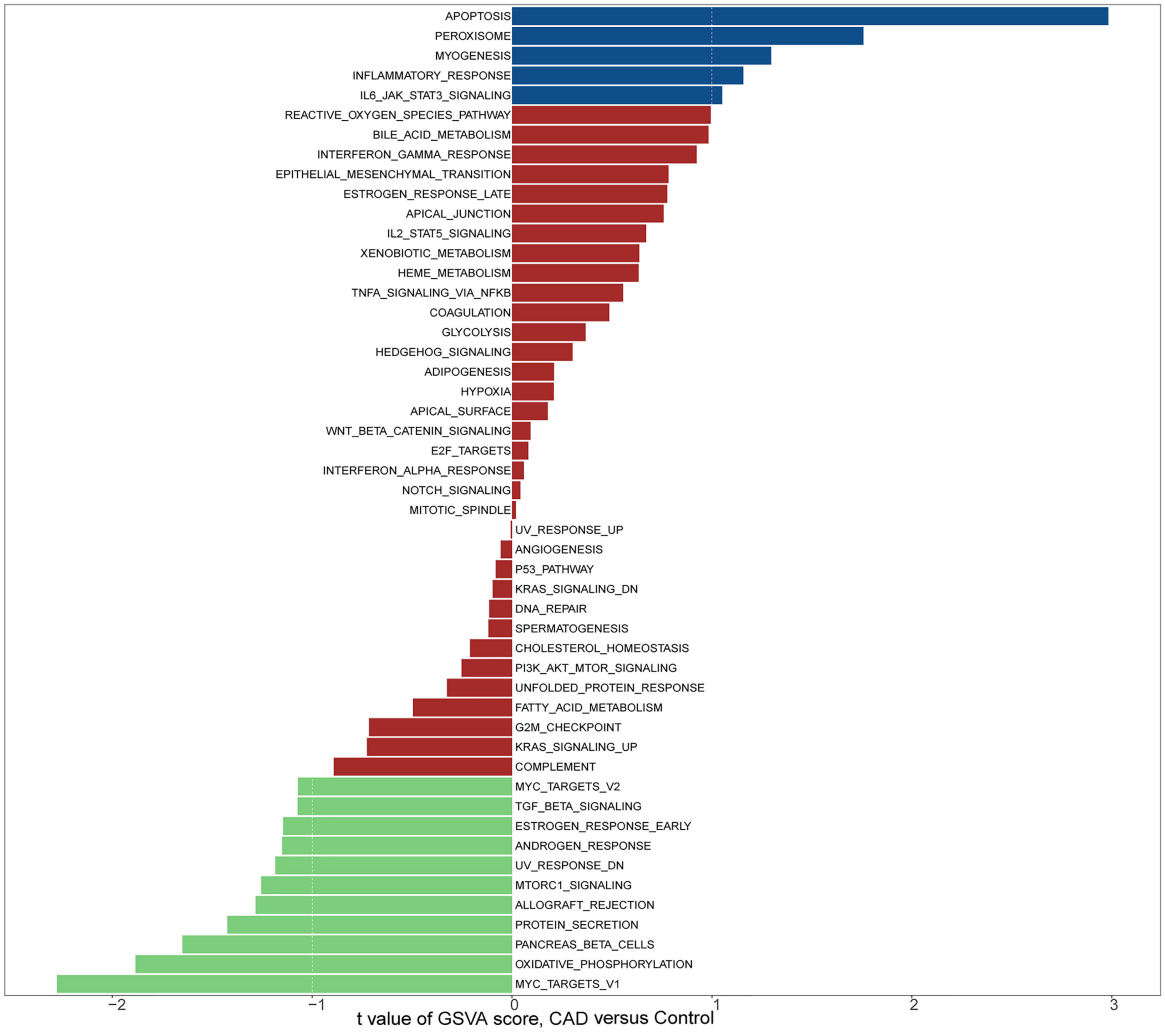
KEGG section of GSVA.

To investigate the potential role of genes in the GSE12288 dataset associated with CAD, we performed a GSVA analysis (**Figures 1**, **2**). These genes were significantly enriched in some immune-related pathways, such as il6 jak stat3 signaling, interferon-gamma response, and il2 stat5 signaling.

### Gene Expression Data for CAD

We obtained microarray expression data for coronary artery disease from the GEO database. We selected 664 genes with coefficients of variation greater than 0.1 for the subsequent analysis step.

### Evaluation of CAD-Infiltrating Immune Cells

We analyzed the corresponding expression data in the dataset GSE12288 using the R program “CIBERSORT” to determine the proportion of different immune cell isoforms in each sample in the dataset. Afterward, the proportions of seven T-cell subtypes in each piece were selected as trait data for WGCNA.

### Gene Co-Expression Network of CAD

**Figure 3:**
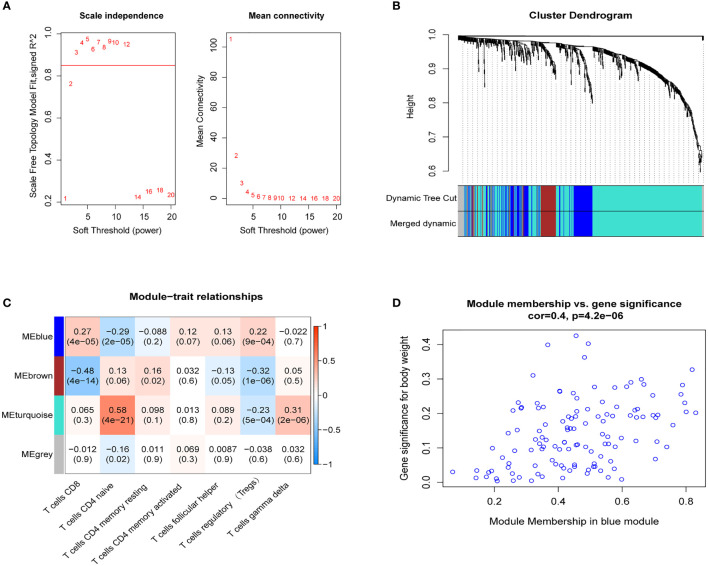
WGCNA. **(A)** Scale-free fit index analysis of 1-20 soft threshold power (left) as well as mean connectivity analysis (right); **(B)** Gene classification into different modules by hierarchical clustering, with different colors representing different modules; **(C)** Heat map showing module features associated with T-cell infiltration; **(D)** Scatter plot of genes in blue modules.

Based on the expression levels of 664 genes in the dataset GSE12288, we constructed a gene co-expression network using the R package “WGCNA.” Then, we calculated the average linkage and Pearson correlation coefficients and performed cluster analysis on all samples in this dataset. We successfully constructed a scale-first network with β=3 as the soft threshold power (**Figure 3**). We also used a dynamic hybrid cut method to build a hierarchical clustering tree. Each leaf represents a gene, and each branch represents a module, which aggregates all genes with similar expression levels. We then summed the functionally equivalent modules into one large module and obtained four modules (**Figure 3**).

### Hub Module Identification and Functional Enrichment Analysis

**Figure 4:**
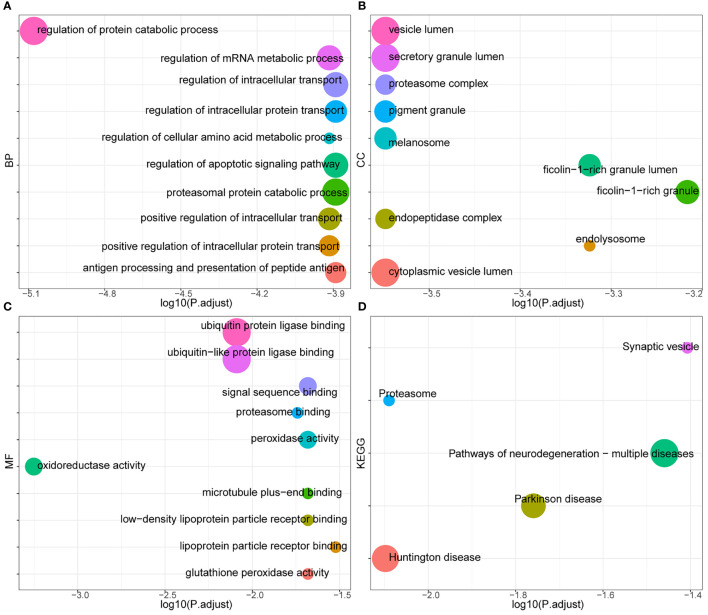
GO and KEGG. To clarify the functions and signaling pathways of genes in the blue module, we performed not only GO analysis containing biological processes **(A)**, cellular components **(B)**, and molecular functions **(C)**, but also KEGG pathway **(D)** analysis.

The blue module of the four modules had the most significant relationship with T-cell CD8 (CD8+ T cells) (R2 = 0.27, P=4E-05; **Figure 3**). Therefore, we defined the blue module as the hub module. In this module, the genes with higher linkage were identified as potentially essential genes most associated with CD8+ T cells. Based on the truncation criteria (module affiliation > 0.8 and gene significance > 0.5), nine genes were selected (PAX8, ATP6V0C, MMP14, PSMF1, RAD23A, PRDX6, MKRN1, YBX3, FBXO7) as candidate hub genes (**Figure 3**). Next, the genes in the blue module were analyzed by GO and KEGG analysis using the “cluster profile” R package. In BP, module genes were significantly enriched in regulating protein catabolic process, cellular amino acid metabolic process, and positive regulation of intracellular transport (**Figure 4**). In CC, modular genes were significantly enriched in proteasome complex, endopeptidase complex, and melanosome (**Figure 4**). In MF, modular genes were increased dramatically in ubiquitin-protein ligase binding, ubiquitin-like protein ligase binding, and proteasome binding (**Figure 4**). In KEGG, modular genes were significantly enriched in Huntington disease, Proteasome, and Parkinson’s disease (**Figure 4**).

### Identification and Validation of Hub Genes

**Figure 5:**
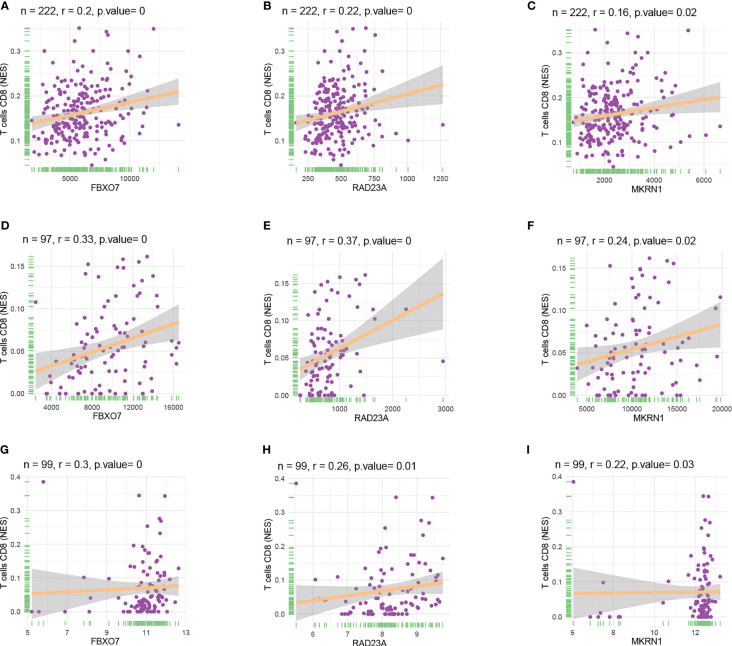
Scatter plots of hub gene expression versus CD8+ T cell infiltration levels in three different datasets: **(A-C)** GSE12288. **(D-F)** GSE34198. **(G-I)** GSE66360.

**Figure 6:**
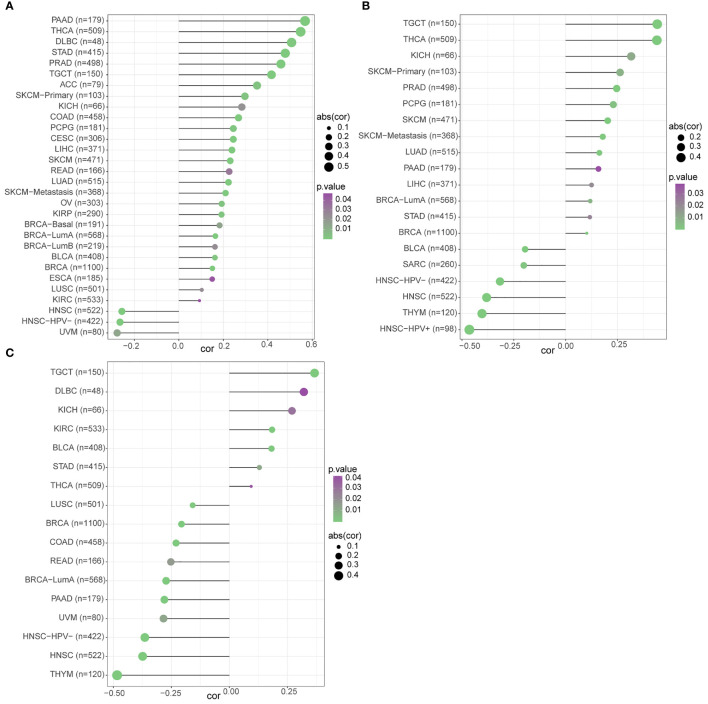
The relationship between the three hub gene expression levels and the degree of CD8+ T cell infiltration in different cancers was statistically significant at P<0.05: **(A)** FBXO7. **(B)** RAD23A. **(C)** MKRN1.

We used three datasets, GSE12288, GSE34198, and GSE66360, to identify and validate the correlation between PAX8, ATP6V0C, MMP14, PSMF1, RAD23A, PRDX6, MKRN1, YBX3, and CD8+ T cell infiltration levels, and finally determined that FBXO7, RAD23A, and MKRN1 could serve as reliable Hub genes. The analysis results showed that all three hub genes, FBXO7, RAD23A, and MKRN1, were significantly and positively correlated with the degree of infiltration of CD8+ T cells in these three datasets (**Figures 5A**). To further explore the expression values of FBXO7, RAD23A, and MKRN1 genes concerning CD8+ T cells in different cancers, the correlations of FBXO7, RAD23A, and MKRN1 expression values with CD8+ T cells in the TIMER database were next obtained and downloaded and visualized using the “ggplot “ R package for visualization. It was found that the expression values of FBXO7 were also significantly and positively correlated with the degree of CD8+ T cell infiltration in several cancers (**Figure 6**), followed by RAD23A (**Figure 6**) and MKRN1 (**Figure 6**). These analyses confirm that the identified HUB genes are closely associated with the level of CD8+ T-cell infiltration and play an essential role in the tumor immune microenvironment.

### Methylation Levels of Hub Genes

**Figure 7:**
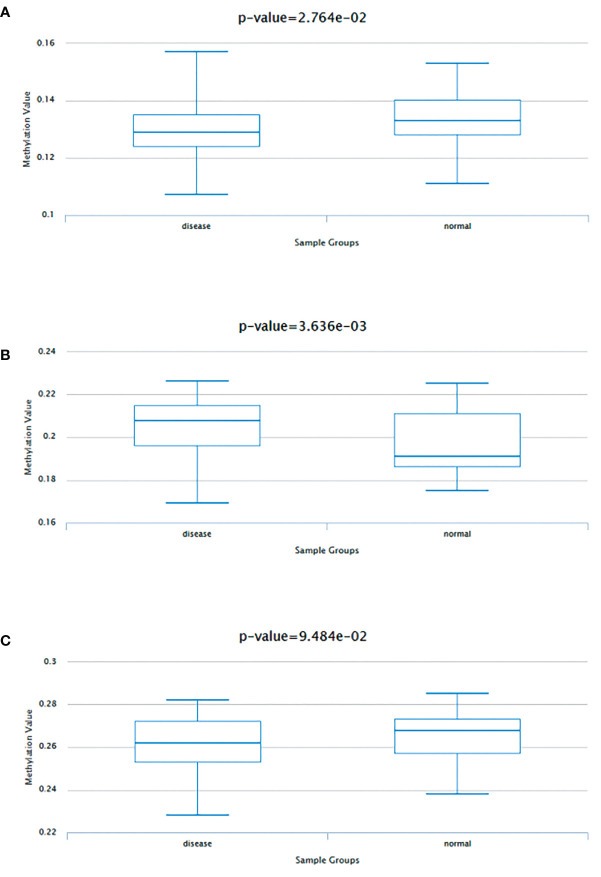
Methylation analysis of three hub genes **(A)** FBXO70, **(B)** RAD23A, and **(C)** MKRN1 in patients with coronary artery disease (left) versus normal subjects (right).

Data consistent with methylation levels of the 3 HUB genes in coronary artery disease were obtained from DiseaseMeth 2.0. analysis showed that FBXO7 showed significantly lower methylation levels (**Figure 7**), and RAD23A had substantially higher methylation levels (**Figure 7**), whereas the methylation results for MKRN1 were not statistically significant (**Figure 7**).

### Hub Gene Correlation

**Figure 8:**
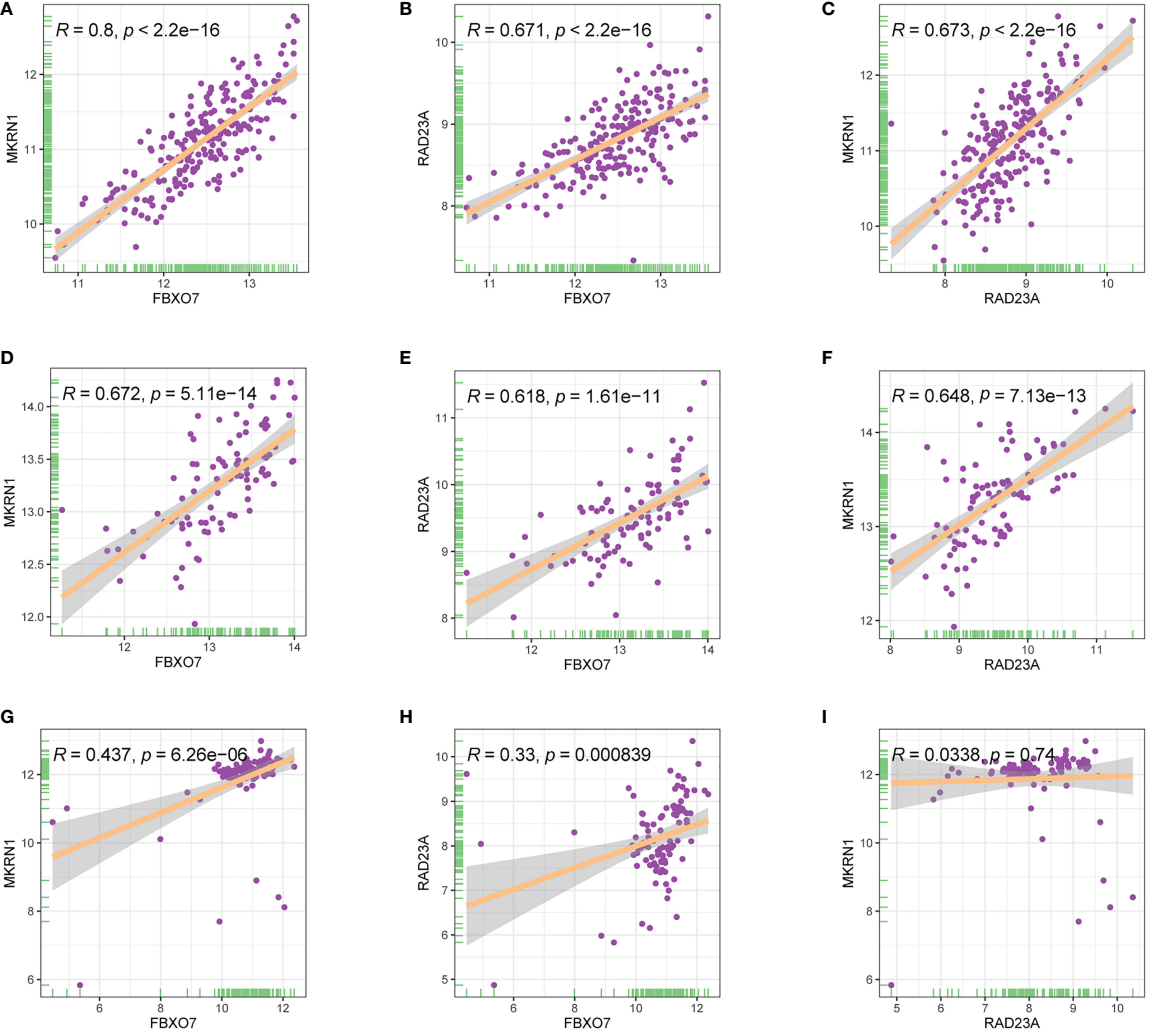
Relationship between the expression levels of three hub genes in three different gene sets **(A-C)** GSE12288. **(D-F)** GSE34198. **(G-I)** GSE66360.

**Figure 9:**
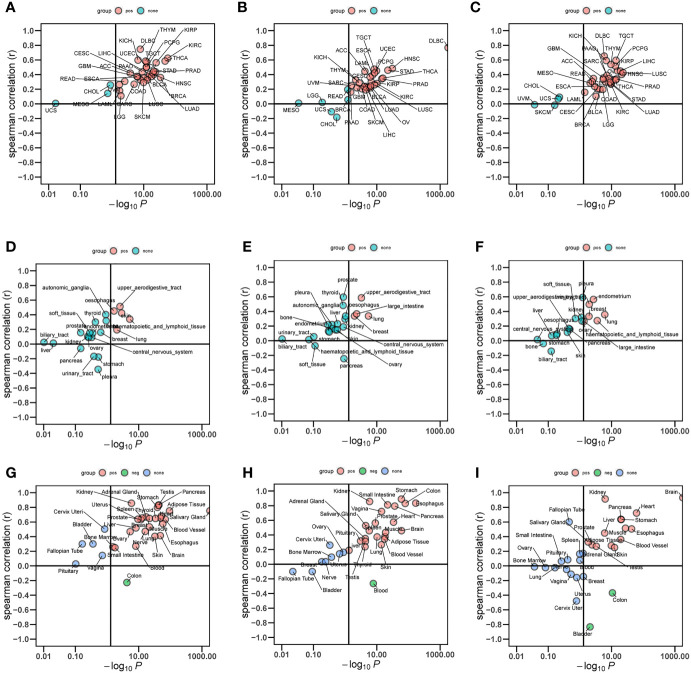
Relationship between the expression levels of three hub genes in different cancer types **(A-C)**, different cell lines **(D-F)**, and normal tissues **(G-I)**.

The analysis showed a significant positive correlation between FBXO7, RAD23A, and MKRN1 in the three datasets GSE12288, GSE34198, and GSE66360 (**Figures 8A**). We next analyzed the correlation between FBXO7, RAD23A, and MKRN1 in different cancers. Except for USC, MESO, and CHOL, FBXO7 was significantly positively correlated with MKRN1 in the remaining cancers (**Figure 9**). MKRN1 was significantly positively correlated with RAD23A in the remaining cancers except for MESO, LGG, UCS, CHOL, READ, and BRCA (**Figure 9**). Except for UVM, CHOL, UCS, SKCM, MKRN1 was significantly positively correlated with RAD23A in the remaining cancers (**Figure 9**). The correlation between FBXO7 and MKRN1 in cell lines is shown in **Figure 9**, the correlation between FBXO7 and RAD23A in cell lines is shown in **Figure 9**, and the correlation between MKRN1 and RAD23A in cell lines is shown in **Figure 9**. FBXO7 was significantly positively correlated with MKRN1 in cardiac tissues (**Figure 9**), FBXO7 was significantly positively correlated with RAD23A in cardiac tissues (**Figure 9**), and MKRN1 was significantly positively correlated with RAD23A in cardiac tissues (**Figure 9**).

### Linkage Between Hub Genes

**Figure 10:**
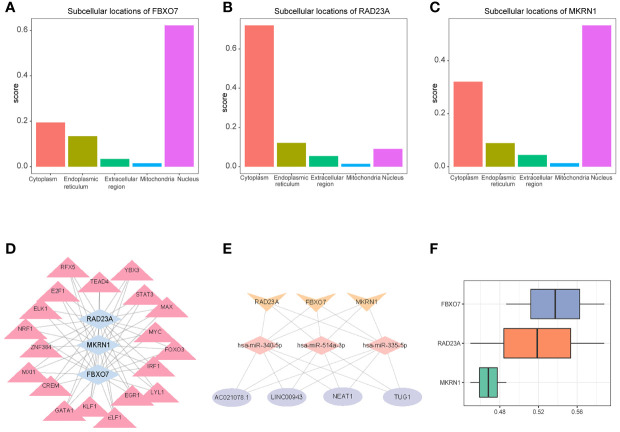
Subcellular localization of 3 hub genes **(A)** FBXO70, **(B)** RAD23A, and **(C)** MKRN1. **(D)** A network composed of target gene transferrin (Gen-TF). The blue diamond is the gene, and the pink triangle is the TF; **(E)** CircRNA-miRNA-Hubgene network. The network consists of four CircRNAs, three miRNAs, and three Hub genes. The yellow V-shape represents mRNAs, the pink diamond represents miRNAs, and the purple circle represents differentially expressed CircRNAs; **(F)** Functional similarity analysis of the three hub genes.

The mRNA subcellular localization results showed that FBXO7 was mainly localized in Nucleus and cytoplasm (**Figure 10**), RAD23A was mainly localized in cytoplasm and Nucleus (**Figure 10**), and MKRN1 was mainly localized in Nucleus and cytoplasm (**Figure 10**). We identified 19 common transcription factors associated with FBXO7, RAD23A and MKRN1 from the CHEA3 database (**Figure 10**). Three common microRNAs associated with FBXO7, RAD23A and MKRN1 (hsa-miR-335-5p, hsa-miR-340-5p and hsa-miR-514a-3p) were identified from the ENCORI database, and common LncRNAs for common microRNAs (AC021078.1, NEAT1, LINC00943, TUG1) (**Figure 10**). The results of functional similarity analysis showed that there were functional similarities among FBXO7, RAD23A and MKRN1, with FBXO7 occupying the most important position (**Figure 10**).

### Small Molecule Drug Analysis

We entered FBXO7, RAD23A, and MKRN1 into the camp database and eventually identified 11 drugs that could be used to treat coronary artery disease, including doxorubicin, gabapentin, suxibuzone, prasterone, kawain, 5255229, decline, nalbuphine, MK-801, trimethoprim, and GW-8510 (**Table 1**).

**Table 1:** hub genes was used to predict potential drugs for the treatment of CAD.

Cmap Name	Mean	N	Enrichment	P	Specificity	Percent Non-Null
doxorubicin	-0.792	3	-0.926	0.00066	0.0827	100
gabapentin	-0.591	4	-0.824	0.00185	0	75
suxibuzone	-0.35	4	-0.815	0.00215	0	50
prasterone	-0.558	4	-0.81	0.00257	0.008	75
kawain	-0.668	5	-0.736	0.00268	0	80
5255229	-0.867	2	-0.942	0.0073	0.0164	100
delsoline	-0.531	4	-0.749	0.00794	0.0226	75
nalbuphine	-0.529	5	-0.677	0.00821	0.0272	60
MK-801	-0.297	4	-0.745	0.00837	0.0108	50
trimethoprim	-0.43	5	-0.674	0.00857	0.0128	60
GW-8510	-0.433	4	-0.74	0.00893	0.2748	50

### Gene Enrichment of GSEA

**Figure 11:**
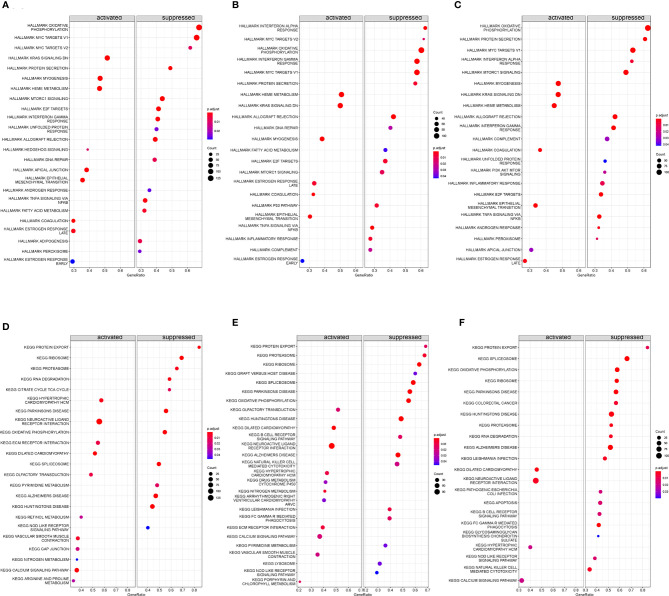
Gene set enrichment analysis (GSEA) was performed to analyze hallmark and KEGG. The entire gene set enriched in samples containing FBXO70 **(A, D)**, RAD23A **(B, E)**, and MKRN1 **(C, F)** was highly expressed.

**Figure 12:**
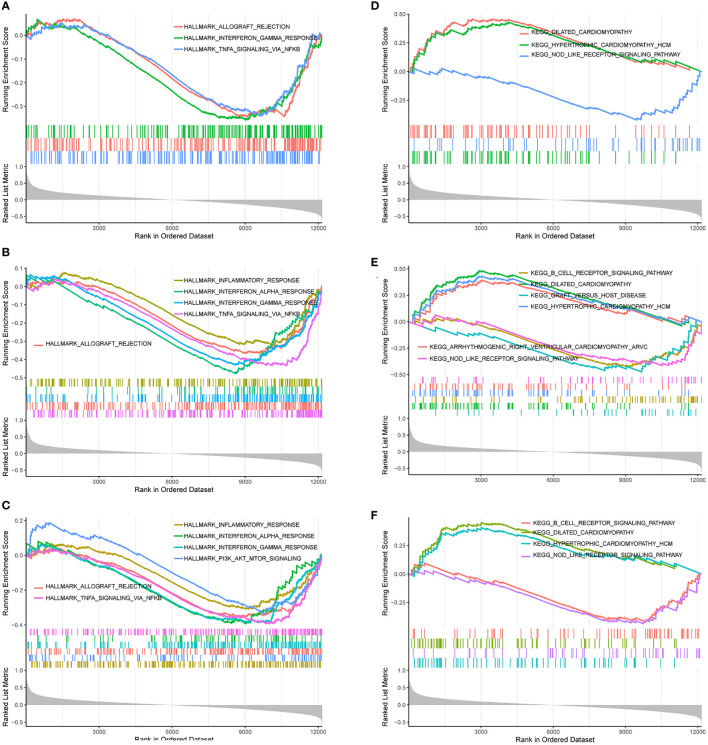
Gene sets are associated with immunity or heart disease. The gene sets related to immunity or heart disease were highly correlated in samples enriched for FBXO70 **(A, D)**, RAD23A **(B, E)**, and MKRN1 **(C, F)**.

Based on “h.all.v7.4.entrez.gmt” as the reference genome, we found the complete list of gene sets enriched in samples related to FBXO7 (**Figure 11**), RAD23A (**Figure 11**), and MKRN1 (**Figure 11**). Based on “c2.cp.kegg.v7.4.entrez.gmt” as the reference genome, we found the complete list of gene sets enriched in samples related to FBXO7 (**Figure 11**), RAD23A (**Figure 11**), and MKRN1 (**Figure 11**). Then, we selected the gene sets associated with immunity and CAD from the complete list for further analysis. When we used “h.all.v7.4.entrez.gmt” as the reference genome, the analysis revealed that the samples of the highly correlated FBXO7 (**Figure 12**) were enriched for allograft rejection, interferon-gamma response, and tnfa signaling *via* NF-kB in 3 sets of gene sets; In contrast, three of the five gene sets enriched in samples from the highly correlated RAD23A (**Figure 12**) were the same as those from FBXO7, in addition to the inflammatory response and the interferon-alpha response; Six gene sets were enriched in the highly correlated MKRN1 (**Figure 12**) samples, except for five of them, the same as the RAD23A enrichment results, and another set of pi3k Akt mtor signaling. And when we used “c2.cp.kegg.v7.4.entrez.gmt” as the reference genome, three sets of gene sets were enriched in the samples of the highly associated FBXO7 (**Figure 12**), including dilated cardiomyopathy, hypertrophic cardiomyopathy hcm, and nod like receptor signaling pathway; Six gene sets were enriched in samples with highly correlated RAD23A (**Figure 12**), three of which were improved in the same way as FBXO7 and the other three in the B cell receptor signaling pathway, graft versus host disease, and arrhythmogenic right ventricular cardiomyopathy arvc; Four gene sets were enriched in the highly correlated MKRN1 (**Figure 12**) samples, except for three of them, the same as the FBXO7 enrichment results and one set of B cell receptor signaling pathways.

## Discussion

Despite the current high level of medical care available, CAD and cancer remain important causes of human mortality worldwide. A growing number of studies show that immune responses are essential in developing both CAD and cancer. It is well known that immunotherapy is an integral part of anti-cancer treatment. Several common types of immunotherapies, such as immune checkpoint blockade (ICB), chimeric antigen receptor (CAR)-T cell therapy, cytokine therapy, immunomodulatory antibodies, monoclonal antibodies, and cancer vaccines, have achieved better anti-cancer responses (32, 33). In contrast, no immunotherapies have been identified that can be applied to the clinical treatment of CAD. In recent years, findings exploring immunotherapeutic approaches for CAD, such as the Canakinumab Anti-Inflammatory Thrombosis Outcome Study (CANTOS) and the Cardiovascular Inflammation Reduction Trial (CIRT), have shown that only blocking specific signaling pathways associated with atherogenesis can be genuinely therapeutic (34, 35). This finding has further stimulated interest in exploring potential target biomarkers for immune correlates of CAD. In addition, patients with cancer combined with CAD may have accelerated CAD progression due to the pro-inflammatory response and hypercoagulability brought about by cancer itself (36). Moreover, anticancer therapy in cancer patients may also increase the risk of developing CAD due to its cardiotoxic effects (37). However, there is little evidence regarding the exact mechanism of interaction between CAD and cancer to date.

This study used WGCNA in the GSE12288 dataset to find the most relevant modules to CD8+ T cells for the following analysis. FBXO7, RAD23A, and MKRN1 were verified and identified as hub genes by using GSE34198 and GSE66360 datasets. Their expression levels were positively correlated with CD8+ T-cell infiltration in CAD and multiple cancers. Among them, FBXO7, as a member of the F-box protein family, can be involved in the pathological process of Parkinson’s disease (PD) development through various mechanisms such as indirect ubiquitination and disruption of autophagic function (38, 39). FBXO7 is a component of the SCF (SKP1/cullin-1/F-box protein)-E3 type ubiquitin ligase complex, which leads to the production of the corresponding substrate ubiquitination (40). Previous correlations have reported that FBXO7 is expressed at high levels in cancer and is a potential oncogene (41). It is associated with immune escape and immunotherapy resistance in cancer patients (42). RAD23A, also known as RAD23 and HHR23A, acts as a junction protein during ubiquitin-mediated proteasomal degradation, increasing the degradation of target proteins and enhancing the response of CD8+ T cells (43). Human tumor virus E6-associated protein (E6AP) acts as an E3 ligase in ubiquitination. HHR23A participates in the ubiquitination process by binding to E6AP (44). rAD23A increases cancer chemoresistance and the chance of recurrence by upregulating autophagy (45). Makorin Ringinger Protein 1 (MKRN1) is an E3-type ubiquitin ligase. It has been shown that MKRN1 promotes p21 protein ubiquitination and proteasome pathway degradation, thereby preventing intermittent hypoxia (IH)-induced myocardial apoptosis (46). MKRN1 can disrupt p53 and thus apoptosis through ubiquitination (47). P53 is known to regulate autophagy, according to previous studies (48). Of course, further experiments are needed to verify whether there is a link between MKRN1, p53, and autophagy. MKRN1 positively regulates biological processes mediated by Wnt/β-linked proteins and plays an essential role in the proliferation, migration, and invasion of cancer cells (49). In addition, it has been reported that MKRN1 may be involved in regulating the development of gastric carcinogenesis (50). depletion of MKRN1 may inhibit tumor growth (51). It has been previously found that ubiquitination is a physiological process by which ubiquitin binds to target proteins to cause a series of reactions in substrates. It plays a crucial role in regulating immune responses (52). Moreover, ubiquitination plays an essential role in the pathogenesis of CAD, and its expression level increases with the severity of CAD (52, 53). On the other hand, autophagy is a conserved evolutionary process that provides energy and biological components by digesting its dysfunctional organelles (54, 55). Early correlations reported that autophagy could be stimulated in surviving cardiomyocytes to produce autophagy for self-preservation in the context of long-term chronic myocardial ischemia (56, 57). In addition, ubiquitination and autophagy, two major pathways of intracellular protein degradation, can be presented to the surface of MCH class I molecules, thereby activating CD8+ T cells and thus inducing an immune response in the organism (58). The ubiquitination of three hub genes, all associated with E3 ligases. Targeting the E3 ligase inhibits tumor progression (59). In the early stages of cancer, autophagy inhibits carcinogenesis development, while it promotes tumor survival and metastasis in later stages (60). Thus, our study provides a potential direction for the involvement of FBXO7, RAD23A, and MKRN1 in the immune response to CAD through ubiquitination or autophagy. Meanwhile, they may play an oncogenic role in cancer and are closely related to CD8+ T cells.

In addition, we found that FBXO7, RAD23A, and MKRN1 were significantly positively correlated in cardiac tissues and most cancers. No correlation studies have been found on the role of these three core genes in the association between CAD and cancer. Our study provides a new direction for the joint research between CAD and cancer while possibly providing possible options for optimizing the treatment of potential CAD in cancer patients. Of course, these need further experimental validation and clinical studies. It has now been confirmed that the efficacy of immunotherapy such as ICB and CAR T-cell therapy for tumors is mainly influenced by epigenetic regulation. And epigenetic modifications associated with CD8+ T cells can enhance immunotherapy (61). As we all know, epigenetics primarily focuses on methylation modifications (62). Therefore, we performed methylation analysis of the three core genes and showed that except for MKRN1, which was not statistically significant, FBXO7 showed hypomethylation, while RAD23A was the exact opposite. This provides a new theory to study the methylation process of FBXO7 and RAD23A and improve potential CAD immune efficacy in cancer patients.

To further understand the linkage of hub genes, we identified 19 common transcription factors associated with FBXO7, RAD23A, and MKRN1 by constructing a TF-gene network. FOXO3 has been reported to be a lifespan-associated gene that increases mortality in elderly patients with CAD (63). At first, FOXO3 was mainly considered a tumor suppressor. However, recent studies have found that it is also involved in maintaining starter cells in leukemia and colon cancer metastasis (64, 65). It was found that blocking STAT3 activity may be an effective strategy for treating CAD (66). STAT3 is often over-activated in various cancers and is associated with tumor immunosuppression and poor prognosis (67). Targeting STAT3 is emerging as a potentially promising therapeutic modality for many cancers (68). These two transcription factors may serve as targets for CAD-cancer interactions. Another 17 transcription factors are not studied for their association with CAD. In addition, there is growing evidence that competitive endogenous RNAs (ceRNAs) play a key role in CAD and cancer (69). However, there is still a lack of ceRNA networks that can predict the diagnosis and treatment of CAD. In the present study, we identified three common microRNAs (hsa-miR-335-5p, hsa-miR-340-5p, and hsa-miR-514a-3p) associated with FBXO7, RAD23A, and MKRN1. It has been suggested that miR-335-5p may regulate the process of cardiomyocyte differentiation by activating the WNT and TGFβ signaling pathways (70). It can be used as a potential biomarker for various cancers, for example, osteosarcoma, breast cancer, and non-Hodgkin’s lymphoma (71–73). In lung cancer, reduced expression of has-miR-340-5p may mediate cisplatin resistance (74). miR-514a-3p promotes melanoma growth (75). All three microRNAs have been studied in cancer in a limited number of ways. However, none of them is associated with CAD so far. At the same time, we also reverse inferred four common LncRNAs (AC021078.1, NEAT1, LINC00943, TUG1) of the common microRNAs. Among them, it was found that NEAT1 may inhibit CAD cell apoptosis by activating the miR-140-3p/MAPK1 pathway (76). neat1 promotes the progression and metastasis of several cancers (77, 78). linc00943 is associated with gastric cancer and clear cell renal cell carcinoma (79, 80). tug1 regulates endothelial cell proliferation and migration through the Wnt pathway to stimulate diabetic atherosclerosis (81). tUG1 is involved in tumorigenesis and tumor progression (82). The ceRNA regulatory network constructed in this study may play a role in CAD and cancer development and progression. The TF-gene and ceRNA network suggests that three hub genes may act as a bridge between CAD and cancer, which of course requires further experimental studies.

Meanwhile, to predict potentially effective therapeutic agents for CAD, we applied the CMAP database to identify 11 therapeutic agents that may reverse abnormally high expression of hub genes associated with CAD. Among them, doxorubicin, although improving prognosis and mortality in cancer patients, increased cardiotoxic effects (83). Gabapentin alleviates the painful reaction caused after coronary artery bypass grafting (CABG) in patients with CAD (84). Delsoline plays an essential role in cardiac remodeling and heart failure progression after myocardial infarction (85). For the 11 drugs identified in this study, no relevant studies have been found so far in cardiovascular disease, except for the above three drugs for which there are few relevant reports. Our findings may provide an essential basis for future exploration of effective drugs for CAD treatment.

In addition to the above studies, to further understand the potential biological pathways between CAD and cancer, we performed GSVA analysis on all samples in the dataset GSE12288. Genes in this dataset were significantly enriched in biological pathways such as il6 jak stat3 signaling, interferon-gamma response (IFN-γ), and il2 stat5 signaling. Single gene GSEA analysis of the screened three hub genes was also performed. The results showed that they were significantly enriched in multiple pathways such as Inflammatory response, interferon-alpha response, interferon-gamma response, TNF signaling *via* NF-kB, allograft rejection, and pi3k Akt mTOR signaling, but also in various cardiomyopathies such as dilated cardiomyopathy and hypertrophic cardiomyopathy. A literature review revealed that flavanones could inhibit the IL6/JAK/STAT3/SOCS3 signaling pathway and improve atherosclerosis (86). The IL6/JAK/STAT3 signaling pathway plays an essential role in the proliferation, survival, and invasion of tumor cells (87, 88). Meanwhile, targeting the IL-6/JAK/STAT3 signaling axis downregulates the expression levels of the CD8+ T cell surface receptors PD-1 or PD-L1 (89). Thus, IL-6/JAK/STAT3 may directly inhibit tumor cell growth or indirectly enhance the antitumor immune response effects of immune checkpoint inhibitors. CD8+ T cells may promote atherosclerosis by increasing the expression level of IFN-γ (11). CD8+ T cells can also induce cytotoxic effects in cancer cells by releasing IFN-γ (90). In the presence of IFN-γ, cancer cells can release several immunosuppressive mediators, including PD-L1 and transcriptional activator 3 (STAT3). These mediators negatively feedback to inhibit the release of IFN-γ, thereby suppressing the activity of CD8+ T cells (91, 92). IL-2 activates STAT5 in CD8+ T cells (93). H9T, an agonist of IL-2, leads to increased expression of CD8+ T cells. In animal models of melanoma and acute lymphoblastic leukemia, CAR-modified CD8+ T cells expanded with H9T have strong antitumor activity (94). The pro-inflammatory cytokine IFN-α can lead to atherosclerosis flare-up and progression (95). the antitumor effect of IFN-α is mediated mainly by CD8+ T cells (96). the inflammatory response has been considered a cancer marker and is closely associated with cancer progression (97). In addition, it is an essential pathogenic mechanism of atherosclerosis (5). MicroRNA-499 was found to cause endothelial cell injury by activating tnfa signaling *via* NF-kB (98). And NF-kB is also involved in tumorigenesis and development (99). Triclosan (TCS) can damage endothelial cells by inhibiting the PI3K/Akt/mTOR signaling pathway (100). The PI3K/Akt/mTOR pathway is usually activated in cancer, and inhibition may have an anti-cancer effect (101). These findings reaffirm that the development of CAD is associated with multiple immune processes mediated by CD8+ T cells. Moreover, these immune processes play a crucial role in cancer.

In conclusion, we identified FBXO7, RAD23A, and MKRN1 as biomarkers in CAD associated with immune responses mediated by CD8+ T cells. This finding helps to understand the part of the immune response in the development of CAD. At the same time, they play the role of potential therapeutic targets in various cancers. FBXO7, RAD23A and MKRN1 may act as hub genes linking CAD and cancer in terms of immunity. It may provide some theoretical basis for identifying new drug targets and developing new therapeutic approaches to reduce the incidence of CAD in cancer patients. Of course, there are some limitations to this study. On the one hand, FBXO7, RAD23A, and MKRN1 have not been reported in CAD. The mechanism of their roles in CAD needs to be studied in depth. On the other hand, the functions of these three hub genes in CAD and cancer are only based on our bioinformatics analysis, and future *in vitro* or *in vivo* experiments are needed to validate these findings further.

## Data Availability Statement

The datasets presented in this study can be found in online repositories. The names of the repository/repositories and accession number(s) can be found in the article/supplementary materials.

## Author Contributions

JZ, YW, XX, YanW, and SZ substantially contributed to conception and design. SZ and YW conducted the literature search. SZ and YW contributed to the acquisition of data or analysis and interpretation of data. SZ wrote the article. YW performed data analysis and drafted. JZ revised the article. All authors gave the final approval of the version to be submitted.

## Funding

This work was supported by scientific research funding from the 2018 Science and Technology Plan Project of the Yunnan Provincial Science and Technology Department. Grant Program [2018FE001 (–285)].

## Conflict of Interest

The authors declare that the research was conducted in the absence of any commercial or financial relationships that could be construed as a potential conflict of interest.

## Publisher’s Note

All claims expressed in this article are solely those of the authors and do not necessarily represent those of their affiliated organizations, or those of the publisher, the editors and the reviewers. Any product that may be evaluated in this article, or claim that may be made by its manufacturer, is not guaranteed or endorsed by the publisher.
